# Alpha Thalassemia/Mental Retardation Syndrome X-Linked, the Alternative Lengthening of Telomere Phenotype, and Gliomagenesis: Current Understandings and Future Potential

**DOI:** 10.3389/fonc.2017.00322

**Published:** 2018-01-08

**Authors:** Jenny He, Alireza Mansouri, Sunit Das

**Affiliations:** ^1^McGill University, Montreal, QC, Canada; ^2^National Institutes of Health (NIH), Bethesda, MD, United States; ^3^St. Michael’s Hospital, Toronto, ON, Canada; ^4^Hospital for Sick Children, Toronto, ON, Canada

**Keywords:** alternative lengthening of telomeres, alpha thalassemia/mental retardation syndrome X-linked, diagnostics, glioma, therapeutics

## Abstract

Gliomas are the most common primary malignant brain tumor in humans. Lower grade gliomas are usually less aggressive but many cases eventually progress to a more aggressive secondary glioblastoma (GBM, WHO Grade IV), which has a universally fatal prognosis despite maximal surgical resection and concurrent chemo-radiation. With the identification of molecular markers, however, there is promise for improving diagnostic and therapeutic strategies. One of the key molecular alterations in gliomas is the alpha thalassemia/mental retardation syndrome X-linked (ATRX) gene, which is frequently mutated. One-third of pediatric GBM cases are also found to have the ATRX mutation and the genetic signatures are different from adult cases. The exact role of ATRX mutations in gliomagenesis, however, is unclear. In this review, we describe the normal cellular function of the ATRX gene product followed by consequences of its dysfunction. Furthermore, its possible association with the alternative lengthening of telomeres (ALT) phenotype is outlined. Lastly, therapeutic options potentiated through a better understanding of ATRX and the ALT phenotype are explored.

## The Alpha Thalassemia/Mental Retardation Syndrome X-Linked (ATRX) Gene and Its Role in Human Disease

The ATRX gene is located on the q arm of the X chromosome. It has been hypothesized that in patients with the ATRX syndrome, both copies are inactivated, one by mutation and the other by X inactivation (1). ATRX mutations result in a truncated protein and, therefore, diminished protein expression. Functional ATRX is one subunit of a chromatin-remodeling complex, forming a heterodimer with death domain-associated protein (DAXX). In oncology, ATRX gene mutations were first discovered in pancreatic neuroendocrine tumors (PanNETs) (2), where mutations of ATRX and DAXX are associated with a better prognosis. Subsequent work has identified these mutations in a range of other cancer subtypes, including gliomas.

## ATRX and DNA Repair

Alpha thalassemia/mental retardation syndrome X-linked is a member of the SWI/SNF family of chromatin-remodeling proteins, with two highly conserved domains (3). The N-terminal ATRX-Dnmt3-Dnmt3L (ADD) domain contains a GATA-like zinc finger, a PHD finger, and an alpha helix. The ATPase/helicase domain is located at the C-terminus. SWI/SNF family proteins disrupt DNA–histone interactions in order to facilitate histone octamer sliding, which changes the conformation of the nucleosome and facilitates chromatin remodeling (4).

Many chromatin-remodeling enzymes have been implicated in human disease (4). Mutations in either of the two ATRX domains are known to cause ATRX syndrome, an X-linked intellectual disability syndrome, although the consequences of mutations in the ADD domain are generally more severe (5). The ATRX gene product interacts with the transcription factor DAXX to form a heterodimer that displays ATP-dependent chromatin-remodeling functions, a non-covalent mechanism that helps regulate gene expression in eukaryotes (6). The ADD domain of ATRX binds histone H3 tail peptides, which contain lysine 9 trimethylation (H3K9me3) marks, while DAXX is a specific chaperone for the histone variant H3.3. Together, the heterodimer facilitates the deposition of H3.3 into telomeric and peri-centromeric chromatin which are rich in H3K9me3 marks (7). ATRX and DAXX localize predominantly in the nucleus and are associated with PML nuclear bodies (8).

The histone variant H3.3 has been implicated in the formation and maintenance of chromatin structure. Nucleosomes containing H3.3 are assembled independent of replication by chaperone proteins, such as DAXX (9). The mammalian genome encodes three different H3 variants. H3.1 and H3.2 are primarily expressed during S-phase of the cell cycle, while H3.3 is replication independent and expressed throughout the cell cycle. H3.3 has previously been found to associate with regulatory elements and heterochromatin at telomeres, and the ATRX/DAXX complex is necessary for its incorporation (7, 10).

Alpha thalassemia/mental retardation syndrome X-linked-Dnmt3-Dnmt3L domain mutations disrupt H3K9me3 binding, which is the general pathway of ATRX mutation pathogenicity. Although the complete function of the ADD domain remains unknown, the PHD finger is known to differentially recognize methylated or unmodified lysine residues in histone tails (11, 12). Reduced ADD binding to H3 tail peptides leads to loss of heterochromatic localization of the ATRX/DAXX complex in cells (5).

Mutations in the ATRX gene lead to changes in methylation patterns of many CpG-rich repetitive sequences, which presents a link between chromatin-remodeling processes and DNA methylation (13). Patients with ATRX syndrome display hypomethylation in subtelomeric regions and rDNA genes, particularly in the CpG-rich regions of the rDNA repeats. Conversely, hypermethylation has been found in the Y-specific repeat (DYZ2) of the Y chromosome. These findings suggest that ATRX may influence CpG-rich regions of the genome and ATRX mutation may have implications on gene expression (13).

Additionally, mutations in SWI/SNF family proteins have been found in approximately 19.6% of human cancers, indicating that SWI/SNF is the most frequently mutated chromatin-regulatory complex in human cancer (14). These proteins have been implicated as tumor suppressors in various tissues; thus, dysfunction may contribute to tumor formation or progression. Various tumor suppression mechanisms have been presented, including the possibility that these complexes contribute to genome stability (15) and influence DNA damage responses (DDRs) (16). One study suggested that SWI/SNF complexes contribute to tumor suppression by activating the DDR and attenuating replicative stress (17). In fact, disruption of both catalytic subunits of SWI/SNF complexes in mouse embryos or D98 tissue-culture cells (cervical carcinoma subline) decreases replication fork efficiency by 50%. Collapsed replication forks highly contribute to genomic instability (18), suggesting the involvement of SWI/SNF complexes in the DDR. SWI/SNF complex deficiency was also found to prevent full activation of DDR kinases such as ATM and ATR, preventing high levels H2AX phosphorylation, an early event in the DDR.

## ATRX in Gliomas

Gliomas represent about 80% of all malignant brain tumors (13) and are classified into four major grades based on their clinical behavior and histology (19). The discovery of ATRX mutations has dramatically influenced the understanding of gliomagenesis.

Data from one study showed that the majority (~75%) of adult lower grade (WHO II/III) gliomas which have p53 and IDH1 mutations also carry ATRX mutations, suggesting an association or a possible driver role in gliomagenesis and progression to secondary glioblastoma (GBM) (20). The incidence of ATRX mutation is higher in diffuse lower grade astrocytomas, compared with oligodendrogliomas and GBMs (2). However, not all oligodendroglial tumors are free of ATRX mutations. Therefore, additional strategies are necessary to delineate whether the cell of origin is oligodendroglial or astrocytic. Given that mutation in the ATRX gene results in a loss of protein expression, immunohistochemical (IHC) analysis should demonstrate a lack of nuclear ATRX expression in tumor cells with mutated/lost ATRX. Reuss et al. have shown a high concordance between retention of nuclear ATRX expression and Grade II/III oligodendrogliomas; a much higher proportion of Grade II/III astrocytomas were found to have loss of nuclear ATRX expression (2). The addition of IHC staining for canonical IDH mutations can help increase the granularity of tumor classification. Tumors with loss of ATRX expression and *IDH*1/2 mutations can be reliably classified as diffuse astrocytomas while *IDH*1/2 mutant tumors with retained ATRX expression should undergo testing for 1p/19q co-deletion to help differentiate between a diffuse astrocytoma and oligodendroglioma (21). In younger patients (age <55 years), given the high prevalence of *IDH* mutations, the absence of mutation detection by standard IHC methods should prompt sequencing for canonical/non-canonical mutations (22).

## The Alternative Lengthening of Telomeres (ALT) Phenotype

The majority of human cancer cells upregulate telomerase to avoid cellular senescence or apoptosis. However, about 10–15% of cancers use an alternative approach to achieve immortality (23). The ALT phenotype is a homologous recombination-associated process but the mechanism is not fully understood. The ALT phenotype is characterized by the presence of ALT-associated promyelocytic leukemia bodies (APBs), which differ from common PML bodies, as well as by telomere recombination with the presence of extrachromosomal telomeric repeats (ECTRs) (24), which may serve as a template for extension of telomeres (25). Recently, it has been shown that many ALT-positive tumors, including PanNETs, oligodendrogliomas, and GBM, contain inactivating mutations in ATRX and/or DAXX, implicating the possible role of ATRX/DAXX inactivation in the manifestation of the ALT phenotype (23, 26).

The ALT phenotype is usually detected using telomere fluorescence *in situ* hybridization, with large, ultrabright signals indicating the presence of ALT (27). Though the ALT mechanism of telomere maintenance is most characteristically found in cancer cells and immortalized cells, some have shown their presence in non-neoplastic cells as well (28, 29). The latter group of cells tended to have a higher rate of DNA damage. Some studies have suggested that the ALT phenotype is not strictly found in ATRX mutant cells and that alteration of other pathways resulting in DNA damage have also been implicated; these include the absence of MYCN amplification and alterations in the TP53 pathway (30). These findings further strengthen the putative association between DNA damage and the ALT phenotype (29). Whether DNA damage is caused by or selects for the ALT phenotype is not clear.

The predominant view of the ALT mechanism is that it maintains telomeres through homology-directed recombination (HDR). ALT-positive cells display a wide range of telomere repeat lengths, increased levels of telomere sister chromatid exchanges (T-SCEs), ECTRs, and the presence of nuclear APBs (31). APBs are nuclear structures that are known to play a role in DNA repair and recombination processes; therefore, it is hypothesized that ALT-mediated telomere elongation takes place within them. In one study performed on PanNETs, a perfect correlation was found between inactivation of ATRX or DAXX and the ALT phenotype (27). The same study also explored the ALT status of CNS tumors with and without ATRX mutations, and a similarly strong correlation was found. Akin to the higher rate of ATRX mutations in lower grade gliomas and secondary GBM, the ALT phenotype was shown to be more common in these tumor groups as well (32).

## ATRX, ALT, and Oncogenesis

Despite the role of testing for ATRX to better refine our ability to characterize astrocytic versus oligodendrocytic glioma lineage, the complete role of ATRX in tumorigenesis remains uncertain. Many studies have implicated ATRX loss in promoting a genetically unstable tumor, with impaired telomere maintenance and disrupted DNA repair, which is more aggressive when left untreated, but also more responsive to double-stranded DNA-breaking agents (20). In fact, recent data have shown that pediatric and adult patients with ATRX mutated GBM have a survival advantage when treated with double-stranded DNA-breaking agents like doxorubicin, irinotecan (SN-38), and topotecan (20). It is possible that the genetic instability is partially a result of ALT activation as the ALT phenotype in other cancer types is associated with genetic instability and altered DNA repair (23).

In addition to binding histone H3 tails, ATRX can also be recruited to G-rich repeats present at telomeres. One study used deep sequencing to show that ATRX preferentially binds tandemly repetitive DNA, especially telomeric G-rich repeats and CpG islands (33). These DNA regions are able to form non-canonical conformations such as G-quadruplexes (G4) *in vivo* (34) and ATRX preferentially binds G4 structured DNA over unfolded sequences (33). Given the association of ATRX with DAXX, the latter is able to enhance H3.3 deposition at these repetitive sequences. Since ATRX preferentially binds these DNA secondary structures, ATRX loss and the presence of abnormal secondary structures may affect the expression of genes near the tandem repeat DNA. G4 structures form barriers to processes like DNA replication and transcription (35, 36), and ATRX loss has been associated with replication defects such as replication fork stalling, prolonged S-phase, accumulation of p53, and H2AX DNA damage marker (37, 38). These problems in replication can cause disruptions of local gene expression and propagation of histone forks during replication. Additionally, increased replication fork stalling, which occurs when functional ATRX is absent, is a known trigger of homologous recombination and can contribute to the activation of the ALT pathway (36). This lends to the hypothesis that stable formation of G4 structures at telomeres impedes replication and triggers the DDR, and functional ATRX (with DAXX and H3.3) may have a role in suppressing ALT pathway activation by binding to G4 structures and resolving them (36).

Additionally, ATRX loss may impair DNA repair. A recent study by Koschmann et al. used a mouse model of ATRX-deficient GBM and found that ATRX loss accelerated GBM growth rate and reduced median survival (39). The mouse brain tumors appeared in the first few weeks of life, similar to the development of pediatric/young adult GBM in humans, which frequently have ATRX mutations (39). They also found that ATRX loss correlated with the ALT phenotype, along with a decrease in expression of non-homologous end joining (NHEJ) repair pathway proteins. Based on the role of ATRX in histone H3.3 deposition, they hypothesized that ATRX loss may lead to conformational changes in heterochromatin that serve to prevent access of NHEJ proteins to the damaged DNA. NHEJ impairment is a mechanism for genetic instability that has been thought to contribute to gliomagenesis, as NHEJ inhibition induces the accumulation of dsDNA breaks (40). A relative increase in HDR compared with NHEJ in ATRX-deficient glioma may play a role in ALT activation.

**Figure 1:**
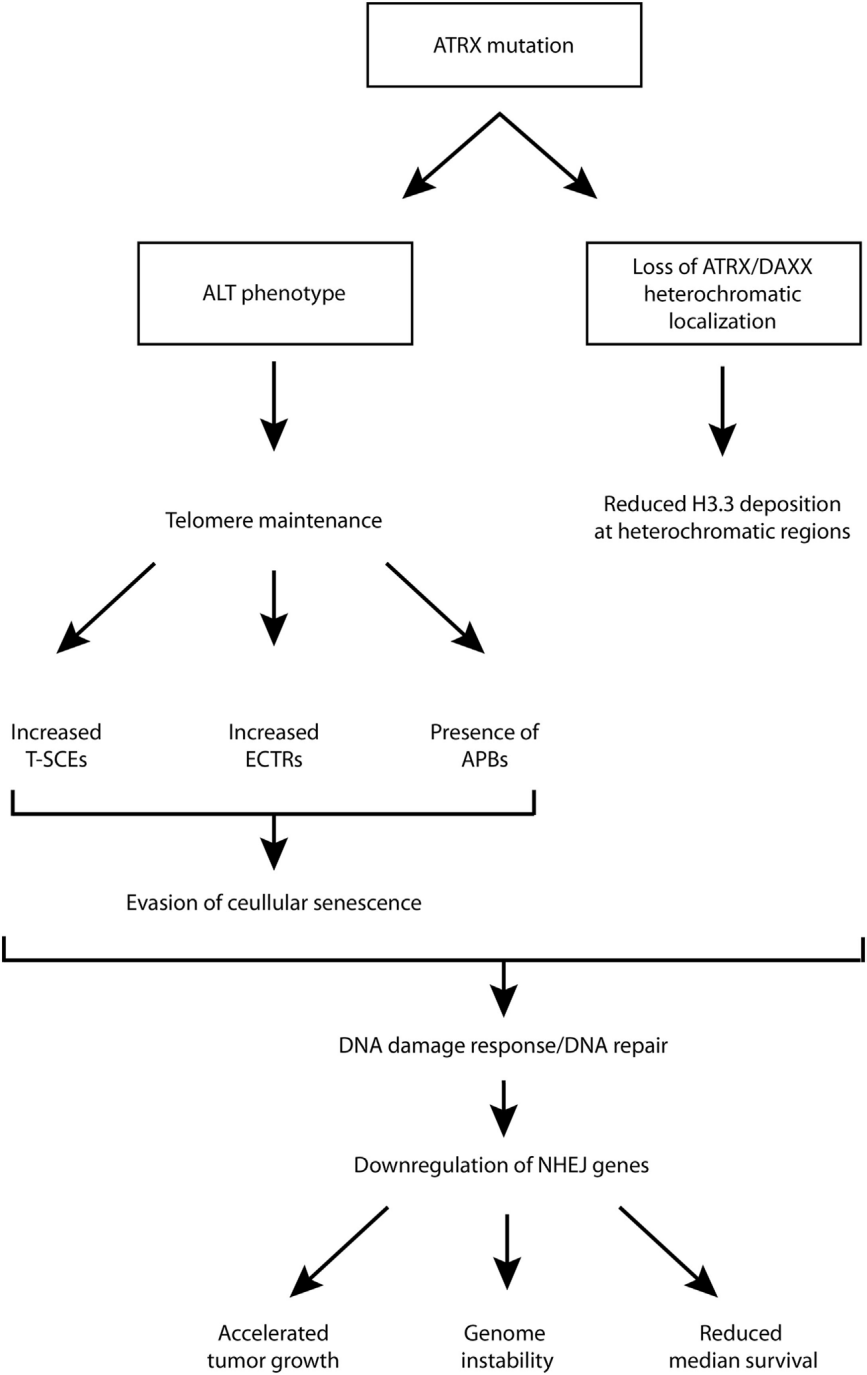
Schematic diagram of putative downstream molecular and cellular events following ATRX mutation. ALT, alternative lengthening of telomeres; APB, ALT-associated promyelocytic leukemia bodies; ATRX, alpha thalassemia/mental retardation syndrome X-linked; DAXX, death domain-associated protein; ECTR, extrachromosomal telomeric repeat; NHEJ, non-homologous end joining; T-SCE, telomere sister chromatid exchanges.

Although ATRX loss has been found to promote some features of the ALT phenotype, and is generally regarded as a hallmark of ALT-immortalized cell lines, it has been shown that ATRX loss is not sufficient for ALT activation, and additional genetic changes are required (39). One study showed that ATRX/DAXX loss and associated lack of H3.3 deposition into telomeric chromatin is crucial and required for ALT but not sufficient, and the ALT phenotype does not arise from a single mutation (40). For example, G2/M checkpoint dysfunction is also required. It is likely that G2/M checkpoint dysfunction is necessary for proliferation despite considerable DNA damage at telomeres (10). Therefore, while there is strong evidence supporting the role of ATRX loss in promoting the ALT phenotype, additional rigorous studies are necessary to establish its contribution to the phenotype and the mechanism(s) through which this is supported. This has implications for diagnostic and therapeutic strategies as well. A schematic diagram, aiming to summarize some of the possible roles of ATRX loss/mutation has been provided in Figure 1.

## Future Directions

Alpha thalassemia/mental retardation syndrome X-linked is mutated in nearly a third of pediatric GBM (26), rarely found in adult primary GBM but common in lower grade adult glioma (WHO 2/3) and secondary GBM (41). However, there currently have been minimal efforts to target ATRX deficiency to treat glioma, mainly due to a limited understanding of the exact role of ATRX in gliomagenesis and disease progression. There are currently no actively enrolling or completed clinical trials based on ATRX status. The development of appropriate animal models would be a necessary first step to investigating this. Furthermore, as the possibility of personalized medicine seems to be more in sight, a comprehensive understanding of ATRX along with its regulators, and downstream targets would enable a broader set of management strategies in children and young adults with gliomas.

Alpha thalassemia/mental retardation syndrome X-linked loss can promote tumor cell susceptibility to DNA damaging agents that induce double-stranded DNA breaks, so the use of agents that induce double-stranded breaks may be more effective in ATRX negative cells (20). Additionally, future work could be aimed at exploring possible mechanisms for selectively targeting the molecular features unique to the ALT phenotype, including the presence of APBs, ECTRs, or increased T-SCE levels.

## Author Contributions

JH and AM performed literature review and analysis and were involved in the writing of the manuscript. SD performed critical analysis and editing of the manuscript.

## Conflict of Interest Statement

The authors declare that the research was conducted in the absence of any commercial or financial relationships that could be construed as a potential conflict of interest.
